# Spoligotype Variation of *Mycobacterium tuberculosis* Strains Prevailing in Korea

**DOI:** 10.1155/2020/8874309

**Published:** 2020-12-31

**Authors:** Seung-Eun Song, Dong Hyeok Kim, Seong-Han Kim, Mi-Sun Park, Sang-Hee Park, Kil-Soo Lee

**Affiliations:** ^1^Division of Bacterial Disease, Korea Centers for Disease Control and Prevention, Osong-eup, Cheongju-si, Chungcheongbuk-Do 28159, Republic of Korea; ^2^Division of Bacterial Disease Research, Korea Centers for Disease Control and Prevention, Osong-eup, Cheongju-si, Chungcheongbuk-Do 28159, Republic of Korea; ^3^Sejong Institute of Health and Environment, 12 Seobukbu 2-ro Jochiwon-eup, Sejong-si, Republic of Korea; ^4^Clinical Research Center, Masan National Tuberculosis Hospital, Gyeongnam, Republic of Korea

## Abstract

Tuberculosis (TB) is an ongoing global health problem, including in South Korea. To manage TB efficiently, it is necessary to understand the epidemiology, transmission route, and characteristics of prevailing *Mycobacterium tuberculosis* strains. In this study, we investigated microevolutions over time in the spoligotype patterns of *M. tuberculosis* isolated from TB patients in Korea. We collected 1,055 clinical *M. tuberculosis* isolates from 16 provinces in Korea from 1994 to 2006 and analyzed them by spoligotyping. We observed 26 subfamilies, including two large predominant families: a Beijing family (72.7%) and the T family (19.1%). Specifically, the abundance of spoligotype SIT269 from the Beijing-like subfamily significantly increased in the 2000s relative to the 1990s in Korea. This study provides an overview of the *M. tuberculosis* genotype trends over time in Korea. These data also indicate that we should consider the influence of the newly growing SIT269 subtype identified in the Beijing family.

## 1. Introduction

In countries with a high incidence of tuberculosis (TB), a national assessment is important to control TB and grasp the trends of epidemic *Mycobacterium tuberculosis* strains. In addition, inquiry into the causes of large-scale TB outbreaks can help identify the etiology of the disease.

Mycobacteria genotyping methods have proven important tools in understanding TB transmission and the epidemiological link [1]. Spoligotyping is a useful molecular epidemiological typing tool for investigating circulating *M. tuberculosis* strains and their evolutionary lineage, monitoring the dynamics of TB epidemics [2, 3]. This method classifies strains based on the polymorphism of the unique spacer sequence between *M. tuberculosis* direct repeat regions. Although spoligotyping analysis has less discriminatory power than other molecular typing tools, such as pulsed-field gel electrophoresis, mycobacterial interspersed repetitive unit-variable number tandem repeat (MIRU-VNTR) typing, and IS6110 restriction fragment length polymorphism (RFLP) analysis, its PCR basis means that it can be tested reproducibly, simply, and quickly [4]. Too high discriminatory power might fail to detect clusters [5]. Therefore, the slightly lower discriminatory power of spoligotyping analysis can be more advantageous for investigating overall circulating cluster trends.

Spoligotyping is optimal for the overall investigation and quantification of TB because it is able to perform comparisons with numerical characters [4, 5]. In addition, it was performed to determine the international *M. tuberculosis* phylogenetic lineage, as there is a global spoligotype database that was previously established [6]. Thus, spoligotyping can provide an overview of *M. tuberculosis* genotype distributions in high-incidence nations.

To date, there exist only two small-scale studies that identified spoligotype patterns in *M. tuberculosis* strains isolated from some provinces of South Korea [7, 8]. Thus, in this study, we investigated the first large-scale TB epidemic in Korea by spoligotyping and aimed to determine the evolutionary and emerging patterns of Korea *M. tuberculosis* strains through the analysis of spoligotype polymorphisms.

## 2. Methods

### 2.1. Study Population and Sample Selection

All clinical mycobacterial isolates were collected from Korean patients with TB from 1994 to 2006. Strain selection and sample size were performed using simple random sampling and a minimum sample calculation formula [9].

The minimum number of specimens required in the 1990s and the 2000s was 364 and 379 cases, respectively, for a total of 11,135 strains stocked in the Korea Institute of Tuberculosis BioBank and isolated from TB patients who registered for treatment at public healthcare centers in 1994, 1998, and 2003–2006. Considering the dropout rate and minimum sample size at the discretion of an experimenter, 1,151 strains were selected for assessment.

In total, 1,151 clinical isolates were classified based on the year of isolation and incidence rate in 16 provinces of Korea, followed by primary culture. Finally, 1,055 cases were selected, except for the strains that failed to yield culture results owing to contamination or the ones that were isolated from outbreaks in community settings, such as schools or nursing homes. A total of 560 (292 (1994) and 268 (1998)) strains isolated in 1994 and 1998 were selected. During 2003–2006, 495 (122 (2003), 132 (2004), 122 (2005), and 119 (2006)) strains were selected.

### 2.2. Spoligotyping and Strain Matching

Total chromosomal DNA of strains was isolated using Genolyse® (Hain Life Sciences) from colonies grown on Middlebrook 7H10 agar (BD Biosciences). Spoligotyping was performed as described by the manufacturer (Ocimum Biosolutions). The binary spoligotype formats were compared with the SITVIT2 database (http://www.pasteur-guadeloupe.fr:8081/SITVIT2_ONLINE/) [6]. In this database, spoligotype international type (SIT) designates an identical pattern shared by two or more patient isolates, whereas “orphan” patterns are applied for a single isolate that does not correspond to any of the strains recorded in the repository database [6]. Data management and statistical analyses were performed using BioNumerics software (version 7.1 program, Applied Maths). Pearson's chi-squared test was used to determine statistical associations between strain types and specific parameters using SPSS software. Odds ratios were calculated with 95% confidence intervals. A *p* value <0.05 was considered as evidence of a significant difference.

## 3. Results

The results of the spoligotype analysis confirmed that the 1,055 *M. tuberculosis* strains isolated in Korea were classified into four main lineages, 20 types of SITVIT subfamilies, and 34 strains belonged to new orphan spoligotypes (Table 1). In this study, a cluster was characterized by two or more patients with the same spoligotype pattern. A total of 980 isolates (92.9%) were grouped into 33 clusters, which included two to 648 isolates (Table 1).

The profile analysis represented two large genomic families, Beijing and the T family. The Beijing family (Beijing type/Beijing-like type), with a distribution of 72.7% (767/1,055), was the most prevalent family, followed by the T family (19.8%, 209/1,055) (Table 1). The Beijing family is part of lineage 2, also known as the East Asian lineage, and the T family belongs to lineage 4, the Euro-American lineage. The pooled incidence rates of other families, that is, Haarlem (H), Latin American and Mediterranean (LAM), S, Ural-1, X (lineage 4), Manu (lineage 1), and Central Asian Strain (CAS, lineage 3), were less than 2% for each (Table 1).

The 706 isolates showed a typical spoligoprofile of the Beijing genotype subfamily, and 61 had abridged Beijing-like spoligoprofiles (Table 2). Beijing type SIT1 was highly abundant (>60%) in the 1990s and the 2000s (Table 2), but did not result in a conspicuous change.

The abundance of Beijing-like type SIT269 increased significantly from 3.2% in the 1990s to 6.9% in the 2000s (*p* value = 0.0062; Table 2). This specific variation in Korea around that period was not seen in other parts of Far East Asia such as China (0.25∼0.69%) [10, 11], Japan (0.33%) [12] (Table 3), and other countries [13].

In contrast, the abundance of the non-Beijing type strains, consisting mainly of the T family, decreased in the 2000s (23.0%) compared to those in the 1990s (31.3%). The T family (including T, T1, T2, T3, T3-OSA, T5, and ambiguous: T3 T2) was also reduced by 6.6%, from 22.9% to 16.3% (Table 2). In the T family, the T1 subfamily abundance decreased from 16.1% to 11.1%, whereas the T2 subfamily abundance decreased from 4.5% to 2.8%. The presumed cause of the decrease in the abundance of the non-Beijing group was spoligotype SIT53 (13.0% to 8.1%, *p* value = 0.0094; Table 2), which is the prototype pattern of the T1 subfamily and is the second most frequent and widely spread worldwide [6]. In China and Japan, SIT53 was the second most distributed, but the proportion was lower than 5% (4.50, 4.73%; Table 3).

The second most commonly observed T family shared type strains was SIT52 of the T2 subfamily, comprising 18 (3.2%) isolates in the 1990s and 12 (2.4%) isolates in the 2000s, respectively (Table 2).

There were 34 previously unreported spoligotypes (orphan) identified using the international spoligotyping database (SITVIT2) [6] in this study (Table 1). Among these, three spoligotypes formed clusters (8 strains), and the other 26 spoligotypes were unique patterns, including three orphan spoligotypes that are genetically close to the Beijing family.

## 4. Discussion

Korea has seen a steady reduction in the number of new TB patients since 1965. In the 2000s, the decline was slow, similar to the global trend [14]. The development of national TB surveillance systems and the expansion of contact investigation are estimated to cause this change in status.

Spoligotype SIT1 is a typical type of the Beijing family and a predominant genotype in Korea, frequently associated with multiple drug resistance and considered hypervirulent [15]. However, some regions, such as Russia and China, reported a controversial relationship between SIT1 and drug resistance [16, 17]. The Beijing family has been spreading from the northern part of China [18] and is isolated at a high rate in Asia, including Korea [7, 8], Japan [12], Taiwan [13] (Table 3), and other Asian and European countries. These nations are historically and regionally related to China. Recently, it has been reported that the proportion of Beijing type strains is increasing in other regions besides Asia [19, 20].

Results from a recent meta-analysis showed that the Beijing family was related to drug resistance, high transmissibility, and unfavorable outcomes [21–23]. The higher rate of genome mutation in the Beijing family has also been proven experimentally [24]. Thus, an increase in the proportion of the Beijing family may present a problem for national TB control.

The T1 subfamily, although its abundance declined in the 2000s, was another peculiarity, with a difference of approximately 8% in abundance compared with China, Japan, and Taiwan (Table 3) [11–13]. This instability of the modern lineages is thought to affect the host adaptive capacity, rapid propagation, and pathogenicity of *M. tuberculosis* [25, 26]. Therefore, further studies on the recent changes in the *M. tuberculosis* genotype are needed to manage TB properly.

The major limitation of spoligotyping is that it cannot distinguish the representative Beijing type SIT1 cluster, so it is necessary to merge other molecular mechanics methods, such as MIRU-VNTR, to compensate for this drawback. Nevertheless, spoligotyping is a method for quickly and easily analyzing the overall epidemiological relationship between currently circulating *M. tuberculosis* strains.

Because the Beijing superfamily is overwhelmingly dominant in Korea, only two nationwide datasets have been reported on the spoligotyping patterns of Korean TB isolates to date [7, 8]. This study is the first large-scale report on the chronological changes in the distribution of *M. tuberculosis* genotypes in Korea. Although *M. tuberculosis* strains distributed in different regions of Korea were collected, the most prominent *M. tuberculosis* genotype from all the regions was the Beijing family. As the highly virulent Beijing lineage has been the most prevalent in Asia for the past few decades, a new TB control strategy is required to reduce the prevalence of the Beijing lineage.

n particular, the abundance of Beijing-like spoligotype SIT269 increased only in Korea. SIT269 strains have a characteristic spoligotype pattern in which direct region loci 1–36 are absent and loci 37–43 are present, unlike SIT1 (loci 1–34 are absent; loci 35–43 are present). Therefore, it is estimated that it originated from the dominant spoligotype SIT1. As the SIT269 type is evenly and widely distributed in Korea regardless of the region and time period, it is difficult to assume that this is an outbreak of a specific type of strain. To confirm the effect of increasing SIT269 abundance, in addition to epidemiological studies, further studies on the detailed classification, persistence, pathogenicity, and propagation of the strain are needed. It is necessary to develop an additional strategy to prevent TB based on the characterization of new propagation strains. By collecting and analyzing various information related to the molecular dynamics of *M. tuberculosis*, predicting the characteristics of TB propagation and the genotypes of strains that may be prevalent in Korea in the near future will help to manage TB more effectively.

This study has some limitations. First, genotyping was performed only through spoligotyping, without comparison with other methods such as the MIRU-VNTR, which has more loci to complete the genotyping results. Second, in the comparison of isolated strains in the 1990s and the 2000s, samples were not collected in consecutive years. Third, since epidemiological investigations were not conducted on all samples, regional outbreak strains were not completely excluded.

## 5. Conclusions

This study reported the first nationwide TB trends in Korea using the spoligotyping method. The Beijing family, having higher transmissibility and hypervirulence, as supported by experimental and clinical evidence, has caused large TB outbreaks in Korea, and newly evolved lineages, such as SIT269, are another source of public health threats. Thus, further studies are recommended to investigate recent evolutionary strains belonging to the modern Beijing family. These results might contribute to the development of new national health policies and TB management strategies.

## Figures and Tables

**Table 1:** Strain distribution according to genomic families and clusters in Korea from 1994 to 2006.

Lineage	Genomic family (no. of subfamilies)	Number of strains (%)	No. of clusters (no. of clustered strains)	The dominant clusters ^†^SIT no.	No. of strains per dominant cluster (%)
Lineage 2	Beijing (2)	767 (72.7)	8 (762)	1	648 (61.4)
Lineage 4	T (6)	209 (19.8)	14 (184)	53	113 (10.7)
	H (2)	12 (1.1)	2 (7)	50	5 (0.5)
	LAM (2)	3 (0.3)	1 (2)	388	2 (0.2)
	S (1)	2 (0.2)	1 (2)	125	2 (0.2)
	Ural-1 (1)	1 (0.1)	—		
	X (2)	4 (0.4)	1 (3)	92	3 (0.3)
Lineage 1	Manu (2)	6 (0.6)	1 (4)	54	4 (0.4)
Lineage 3	CAS (2)	4 (0.4)	—	—	—
Unknown	New (—)	13 (1.2)	2 (8)	773 (5)	5 (0.5)
Not defined	Orphan (—)	34 (3.2)	3 (8)	K7 (3), K25^*∗*^ (3)	3 (0.3), 3 (0.3)
Total		1055 (100)	33 (980)		788 (74.7)

^†^SIT, shared international type from international spoligotype database SITVIT2 (http://www.pasteur-guadeloupe.fr:8081/SITVIT2).^*∗*^Provisionally numbered unique orphan strains in this study.

**Table 2:** Spoligoprofiles of the Beijing and T families in Korea from 1990s to 2000s.

Subfamilies	SIT no.	1990s (1994, 1998)		2000s (2003–2006)		*p* value^*∗*^
		No. of strains	%	No. of strains	%	
Beijing	1	337	60.2	311	62.8	0.3776
Beijing	190	17	3.0	20	4.0	0.3760
Beijing-like	269	18	3.2	34	6.9	0.0062
Beijing-like	406	3	0.5	6	1.2	—
Beijing	255, 265, 541, 621, 796, 1168, 1311, 1364, 2610	11	2.0	10	2.1	—
T	102	2	0.4	5	1.0	
T1	53	73	13.0	40	8.1	0.0094
T2	52	18	3.2	12	2.4	0.4410
T3-OSA	627	6	1.1	3	0.6	—
T, T1, T2, T3, T5, ambiguous: T3 T2	7, 37, 44, 51, 73, 78, 167, 172, 240, 285, 291, 393, 395, 444, 498, 516, 804, 853, 875, 888, 956, 966, 1214, 1223, 1252, 1597, 1622, 1916, 2538, 2867, 3254, 3283	29	5.2	21	4.2	—

Total		514	91.8	462	93.3	

^*∗*^Tested by Pearson's chi-squared test, *p* < 0.05.

**Table 3:** Comparison of dominant subfamilies from the present study with results from three Asian countries.

Country	Study period	No. of strains	Dominant subfamilies	Dominant SITs^*∗*^	%	References
South Korea	2003–2006	495	Beijing	1	62.83	This study
			Beijing	190	4.04	
			Beijing-like	269	6.86	
			T1	53	8.08	
			T2	52	2.42	

China	2005–2007	2,346	Beijing	1	68.24	[11]
			Beijing	190	1.74	
			T1	53	4.50	
			T2	52	1.53	
			H3	50	1.44	

Japan	2002–2007	909	Beijing	1	73.49	[12]
			T1	53	4.73	
			T2	52	1.54	
			T3-OSA	627	2.42	
			Unknown	2124	1.43	

Taiwan	2002–2004	356	Beijing	1	49.43	[13]
			T1	53	2.24	
			EAI2_Manila	19	8.99	
			H	742	2.52	
			H3	50	5.90	

^*∗*^SITs = spoligotype (or shared) international types; H = Haarlem; OSA = Osaka; EAI = East-African Indian.

## Data Availability

The data can be made available on request to e-mail: roadonly@korea.kr.
